# Quantifying the anisotropy and tortuosity of permeable pathways in clay-rich mudstones using models based on X-ray tomography

**DOI:** 10.1038/s41598-017-14810-1

**Published:** 2017-11-01

**Authors:** Nils R. Backeberg, Francesco Iacoviello, Martin Rittner, Thomas M. Mitchell, Adrian P. Jones, Richard Day, John Wheeler, Paul R. Shearing, Pieter Vermeesch, Alberto Striolo

**Affiliations:** 1 University College London, Department of Earth Sciences, London, WC1E 6BT UK; 2 University College London, Electrochemical Innovation Lab, Department of Chemical Engineering, London, WC1E 6BT UK; 3 Halliburton, Chiswick Park, London, W4 5YE UK; 4 University of Liverpool, Department of Earth, Ocean and Ecological Sciences, Liverpool, L69 3BX UK

## Abstract

The permeability of shales is important, because it controls where oil and gas resources can migrate to and where in the Earth hydrocarbons are ultimately stored. Shales have a well-known anisotropic directional permeability that is inherited from the depositional layering of sedimentary laminations, where the highest permeability is measured parallel to laminations and the lowest permeability is perpendicular to laminations. We combine state of the art laboratory permeability experiments with high-resolution X-ray computed tomography and for the first time can quantify the three-dimensional interconnected pathways through a rock that define the anisotropic behaviour of shales. Experiments record a physical anisotropy in permeability of one to two orders of magnitude. Two- and three-dimensional analyses of micro- and nano-scale X-ray computed tomography illuminate the interconnected pathways through the porous/permeable phases in shales. The tortuosity factor quantifies the apparent decrease in diffusive transport resulting from convolutions of the flow paths through porous media and predicts that the directional anisotropy is fundamentally controlled by the bulk rock mineral geometry. Understanding the mineral-scale control on permeability will allow for better estimations of the extent of recoverable reserves in shale gas plays globally.

## Introduction

Shale gas has received much attention in recent years due to the accessible energy reserves stored in low-permeability organic-rich mudstones and shales. In contrast to conventional oil and gas reservoirs, where the fluids have migrated away from their source rock into structural and lithological traps, the hydrocarbons in unconventional shale gas plays remain trapped within their source rock. Intervals of mudstones and shales with organic matter contents of over 2% matured to the gas pressure-temperature window have the potential for economic shale gas production^1^. Resource estimates of shale gas reservoirs are difficult to predict and the technically recoverable gas is highly dependent on the rock mechanical and fluid properties at depth^2,3^. Shale gas can be extracted by hydraulic-fracturing of the source rock with over-pressured fluids and proppants, which induce an open fracture network around the borehole to stimulate flow of the trapped gas by increasing permeability^4–7^. During stimulation of the reservoir, the interconnected induced and natural fracture network captures the directly accessible gas from the distributed pore network^8^. Thus far, the available production data of stimulated wells show decline curves with low-rates of recoveries after peak production that span up to a decade^9–11^, and possibly beyond. The decline curve has predominantly been studied from the perspective of fracture interconnectivity and fracture closure with time^12^. However, research has already provided much insight into the hierarchical permeability structure related to pore sizes, which can shed further light onto extraction potential of shale gas plays.

Gas is stored as compressed free gas in physical micro- and nano-pores, adsorbed to clays and kerogen, and can be dissolved in kerogen^13–16^, all of which provide the permeable phases through or along which gas can migrate^11,17–19^. The effective permeability through shale gas plays can be defined by the combination of hydraulic and diffusive flow mechanisms through the combined porous and permeable phases^18,20–22^. Due to the tightly packed nature and low permeability of unconventional gas plays, the permeable pathways for stored gas are defined by a complex network of micro- and nano-pores in the organic and inorganic matrix^23–25^. Gas flows through shales by a combination of convection (Darcy flow) and Knudsen diffusion through open pore space, and adsorption, desorption and surface diffusion along the pore walls^11^. The contribution of each mechanism will vary based on the relative size contribution of pore spaces and interconnectivity through the shale. Flow through the pore network is further complicated by the possible presence of fluid mixtures with different viscosities, which can decrease the effective permeability of each fluid based on the fluid’s volume proportion^26–28^.

Open fractures provide an order of magnitude higher permeability and the effective permeability of stimulated shales at reservoir conditions is, at first, dominated by Darcy flow^19^. Darcy flow fails in smaller pore spaces as the diffusion flow mechanisms associated with pore-wall interactions become dominant^29^. The recorded decline curve is probably only best defined by the fracture permeability during the early stages of production as the immediately accessible fracture-captured gas escapes. This is followed by a chemical dis-equilibrium and pressure gradients on the mineral scale that drive diffusion and nano-darcy permeability through the matrix^14,29^. Therefore, we believe that the longevity of the decline curve records the inter-fracture matrix permeability and connectivity to the fracture network within the stimulated area.

The geometry of matrix permeable pathways in a rock is a function of the mineralogy and rock texture. The term shale refers to laminated mudstones with variable mineral proportions of clays, quartz, feldspar and carbonates with diverse minor and trace minerals. Clay-rich “shales” (here defined by ≥60% clay volume) show permeability values in the range of 10^−22^ and 10^−19^ m^2^ (0.1 and 100 nano-darcy, respectively), where the range represents an inherited anisotropy in directional permeability along (higher flow) or across (lower flow) the sedimentary laminations^30,31^. In a different setting, clay-rich fault gouges have a well-developed foliation defined by the parallel alignment of clay minerals. Experiments show a two to three order of magnitude difference in directional permeability across and along the structural clay foliation^32,33^ within the same nano-darcy range reported for shales. This link between structural and sedimentary fabrics suggests that the details of the mineral-scale clay geometry is a key controlling factor in predicting the directional permeability of gas through the rock matrix^34^.

Permeability through shales can been measured in laboratory experiments, but because of the low signal to noise ratio in ultra-low permeability systems it is inherently difficult to accomplish. Accordingly, research has focussed on reconstructing three-dimensional volumes to map the pore space distribution and simulate permeability using computational fluid dynamics, such as the Lattice Boltzmann Method^11,35,36^. Accurate three-dimensional representations of shales require high-resolution imaging capable of characterising the nano- and micro-pore space distributions. Focused ion beam scanning electron microscopy (FIB-SEM) has been used to image nano-scale pores in kerogen at a 12 nm resolution^37^. This method sections the sample and sequential SEM images are stacked together to build a three-dimensional reconstruction, typically applied to very small sample volumes below 20 *μ*m^3^. X-ray computed tomography (X-ray CT) has been successfully used on samples with a resolution of 7 *μ*m to 7 nm, with larger sample sizes coming at a cost of resolution^24^. One of the key advantages of X-ray CT is a non-destructive three-dimensional investigation of samples that captures the real nature without a simulation process, which at a scale below the sectioning in FIB-SEM is somewhat artificial. A detailed review on shale gas advances and challenges emphasised the importance of bridging fluid dynamic scales from nanometer pores in kerogen to entire shale gas reservoirs to allow for representative simulations to be developed^38^. In order to further advance representative models of gas flow through shales, it is necessary to build a better understanding of the mineral phase distribution, associated pore volumes and their interconnectivity in natural systems.

In this study, we characterise the geometry of available permeable pathways as the three-dimensional porous phase (pores + organic matter) volume distribution using high-resolution X-ray CT and SEM imaging techniques and model the relative directional flow using “tortuosity factor”. The tortuosity factor and tortuosity are two different parameters, both characterising the geometry and length of interconnected phases. In porous media, tortuosity (*τ*, as used here) is defined as the ratio of the actual length of the flow-path divided by a straight line length in the direction of flow, which has been used to quantify flow along convoluted pathways^39–41^. The same word is used with different definitions by different authors^42^, for example Costa uses *τ*^2^ for tortuosity^43^; Bear & Bachmat and Berg use *τ*^−1^ for tortuosity^44,45^. It is important to check the definitions when comparing different works, especially as some conceptual confusions have arisen^46^.

Tortuosity factor (τ^*^, as used here) quantifies the apparent decrease in diffusive transport resulting from convolutions of the flow paths through porous media^46–48^. Tortuosity factor includes changes in the cross-sectional area over the finite length of the interconnected flow paths and is better suited for modelling more complex pore networks^46^. Tortuosity factor and tortuosity both scale up proportionally with more tortuous pathways. In a system where the cross-sectional area of the flow path remains constant, tortuosity factor is equal to the square of tortuosity^47^. Both tortuosity and tortuosity factor tend to 1 as the flow pathways become more direct across the volume^48^.

We combine the modelled results with experimental permeability tests to account for the anisotropic permeability behaviour of a clay-rich shale gas play. Samples were prepared from a set of four shale cores collected at ~3700 metres depth within a prospective shale gas interval from two boreholes within the same anonymous basin in Europe (Table 1). Due to the anonymity of the sample location, this contribution focusses on the characterisation of the shale samples with respect to the geometric control of mineralogy on permeability.

**Table 1:** QEMSCAN mineralogy and rock physics results for sample suite.

Shale sample	#1	#2	#3	#4
Borehole	A	A	B	B
Depth (m)	3721	3731	3690	3703
Density (g/cm^3^)	2.41	2.52	2.52	2.60
**Mineralogy** (**volume %**)				
Clays		62.2		61.3
Quartz		11.2		9.6
Plagioclase		2.9		3.6
Dolomite		2.9		9.6
Calcite		0.6		1.3
Muscovite		6.3		5.6
Pyrite		1.3		1.6
Trace minerals		11		4
Porosity + OC _*QEMSCAN*_		1.5		3.4
**Rock physics tests**				
Porosity (%) _*He*–*injection*_	5.6	4.9	2.2	2.7
Effective pressure (MPa)	5	5	5	5
k_*v*–*water*_ (m^2^)	3.2 × 10^−22^	3.5 × 10^−22^	2.5 × 10^−22^	4.0 × 10^−22^
k_*v*–*argon*_ (m^2^)	—	—	1.9 × 10^−22^	2.8 × 10^−22^
k_*h*–*water*_ (m^2^)	—	—	1.6 × 10^−21^	6.3 × 10^−20^
k_*h*–*argon*_ (m^2^)	—	—	9.1 × 10^−22^	2.0 × 10^−20^

QEMSCAN porosity results includes organic carbon (OC). Permeability reported for lamination-perpendicular (k*v*) and lamination-parallel (k*h*) flow.

## Methods

### QEMSCAN

Quantitative Evaluation of Minerals by SCANning electron microscopy (QEMSCAN) is a Scanning Electron Microscope (SEM) technique that combines Back-Scattered Electron (BSE) information with Energy-Dispersive X-Ray Spectroscopy (EDS)^49^. The samples are polished and carbon-coated and BSE intensity and EDS spectra are acquired at a fixed spacing of 1 *μ*m or greater. The effective excitation volume for each measurement depends on acceleration voltage and mineral phases, but is typically in the range of few 100 nm wide and deep into the material. The BSE signals and EDS spectra are matched against a fine-tuned reference mineral database for each acquired pixel, resulting in mineral phase maps of the sample surface. In this study, whole samples were scanned at 10 *μ*m spacing, with smaller detail scans at 1 *μ*m resolution. The scanning resolution limits the minimal size of recognised phases, shapes and connectivity information. Porosity is determined from “background” scanning results, which combine organic matter and physical pore spaces.

### X-ray Computed Tomography

Cylindrical pillars were prepared from the samples using an A Series/Compact Laser Micromachining System (Oxford Laser)^50^. The three-dimensional microstructure of shale samples were investigated using two X-ray microscopes (Carl Zeiss X-ray Microscopy Inc., Pleasanton, CA): micron-scale Zeiss Xradia 520 Versa and nano-scale Zeiss Xradia 810 Ultra 810.

Micro-CT was performed using the Versa 520 platform, which utilises an optically coupled two-stage magnification system. During imaging, a total of 2801 radiographs were acquired over a 360° sample rotation range with an exposure time of 18 seconds per radiograph. The shale sample was placed between the X-ray source and a 2k × 2k detector with a source-to-detector distance of 39.9 mm providing a voxel resolution of ~880 nm using the 4x objective magnification in binning 2 mode. The instrument was operated at 50 kV and 80 *μ*A, employing a low energy filter to optimise transmission and contrast to noise ratio.

Nano-CT was conducted using the Ultra 810 instrument, which employs post-transmission Fresnel zone plates to achieve resolution in the sub 100 nm range. The microscope can operated in high-res and large field of view mode with achievable voxel resolution of 16 and 64 nm respectively. The system also allows absorption and Zernike phase contrast capabilities to leverage non-invasive imaging of a variety of materials at the nano-scale. The use of these systems in tandem enables multi-scale insight into the microstructure of shale samples. For the nano-CT experiments, both absorption-contrast and phase-contrast images of the shale micro-pillar were acquired in the “large field-of-view” (LFOV) mode. A total of 901 projections were collected per 180° sample rotation with an exposure time of 15 seconds for absorption and 30 seconds for phase contrast imaging. This yielded two sets of raw image data, both with an isotropic voxel resolution of 126 nm using a detector pixel binning of 2. The two datasets were then merged at 50% proportions to maximise the signal to noise ratio and to leverage the edge enhancement of the phase contrast image with the density information of the absorption contrast scan^51^.

The raw transmission images from both micro-and nano-scale CT imaging experiments were reconstructed using a commercial image reconstruction software package (Zeiss XMReconstructor, Carl Zeiss X-ray Microscopy Inc., Pleasanton, CA), which employs a filtered back-projection algorithm. The reconstructed grayscale 3D image volumes were subsequently segmented using the Avizo software package (Avizo 9.0, FEI Visualization Sciences Group, Mérignac Cedex, France).

### Rock mechanics

The four shale gas play samples were prepared for porosity and permeability analyses in the Rock and Ice Physics Laboratory at University College London. We prepared a sample of a known volume for porosity measurements using He-injection (AccuPyc II 1340 Pycnometer, Micromeritics Instrument Corp.). Drill cores of 20 mm diameter by 20 mm length were prepared from the four core samples for permeability experiments. For samples 1 and 2, only cores drilled perpendicular to lamination were successfully prepared, whereas both lamination-parallel and perpendicular cores were prepared from samples 3 and 4. The prepared cores were either saturated in deionised and distilled water under vacuum for three days before tested for water permeability (samples 1 through 4), or dried at 60 °C at a minimum of three days until tested for argon permeability (samples 3 and 4). The samples were placed into a PVC jacket in between porous alumina spacers before being loaded into the permeameter, following the analytical set-up for permeability measurements outlined in Mitchell and Faulkner^52^. Permeability was measured at a low effective pressure of 5 MPa, with the confining pressure of 10 MPa and the pore pressure of 5 MPa. We used the pore-pressure oscillation technique to measure permeability, where the sinusoidal pore pressure signal of the upstream side of the sample is compared to the response signal on the sealed downstream side^53,54^. The response signal is reduced in amplitude and offset in phase. The attenuation and phase lag are calculated from the upstream and downstream signals. From these two numbers we calculate the two dimensionless parameters of Fischer and Paterson^55^. For precision and computational efficiency we reduce their two simultaneous equations to one and then solve that numerically. Permeability is then calculated from these dimensionless parameters. Oscillations were set at 1 MPa amplitudes over 4 hour periods and the experiments ran for over 4 days per sample to allow for further saturation. Permeability was continuously measured throughout the experiment and we report the average permeability calculated after saturation. One lamination-parallel core from sample 4 split along lamination and we analysed this sample to simulate the (un-propped) fracture permeability. During the fracture permeability experiments we increased the confining pressure incrementally at 10 MPa ramps up to an effective pressure of 45 MPa, which corresponds to a typical reservoir pressure condition of shale gas plays.

## Results

**Figure 1:**
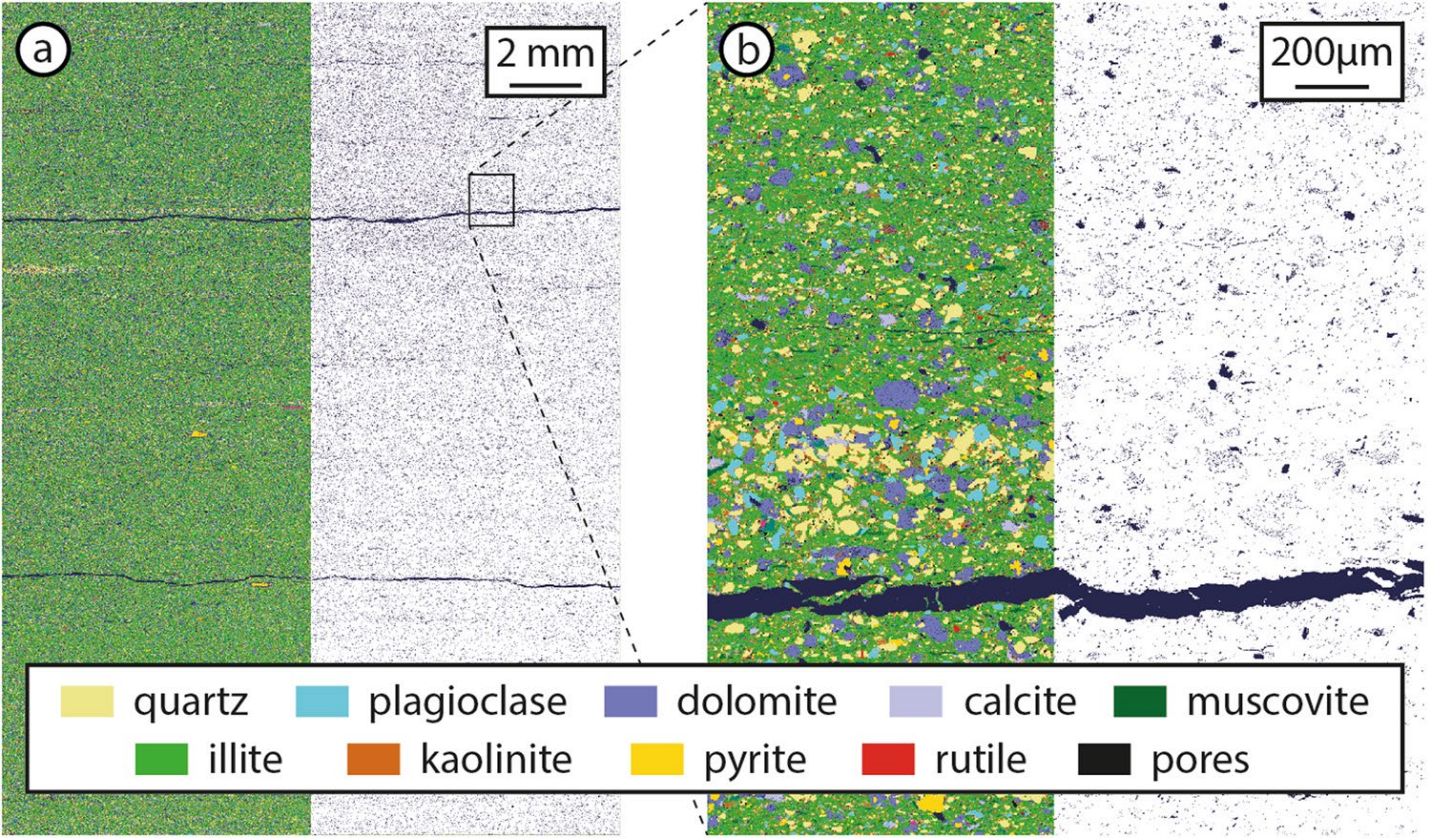
QEMSCAN chemical maps of sample 4 analysed at 10 micron spacing resolution scan of entire thin section (**a**) and 1 micron spacing detailed map (**b**). On the right half of each image we show only pore space distribution on white background. Lamination-parallel fractures visible at both scales. Mineral proportions are reported in Table 1.

The samples analysed in this study are very thin laminated mudstones, or clay-rich shales. QEMSCAN analyses of samples 2 and 4 show ~60 volume % clay minerals (Fig. 1) with a clay composition of 80% of illite, 14% kaolinite and minor smectite. The silt fraction is predominantly composed of quartz, plagioclase and dolomite with trace calcite, pyrite and accessory minerals (Table 1). Muscovite, which is chemically nearly identical to illite, is also reported in the QEMSCAN results, where the grain size of an homogenous cluster of data points was larger than clay minerals. Macroscopic laminations are defined by a combination of silt-rich and clay-rich layers (Fig. 1).

The porosity measured by helium-injection ranges from 2.2% to 5.6%, whereas pore volumes from QEMSCAN analyses are 1.5% for sample 2 and 3.5% for sample 4 (Table 1). Although QEMSCAN likely underestimates the porosity of the samples, the difference between QEMSCAN and He-injection may also be due to sample heterogeneity and varying fracture densities. On each chemical map the distribution of pores is relatively homogenous, diffuse and predominantly disconnected with interconnected pores aligned along fractures and micro-fractures (Fig. 1). The observed fractures are all parallel to laminations, mostly discontinuous and spaced at 0.1 to 2 mm. The large continuous fracture in sample 4 is spatially associated with a thin silt-rich layer (Fig. 1b).

**Figure 2:**
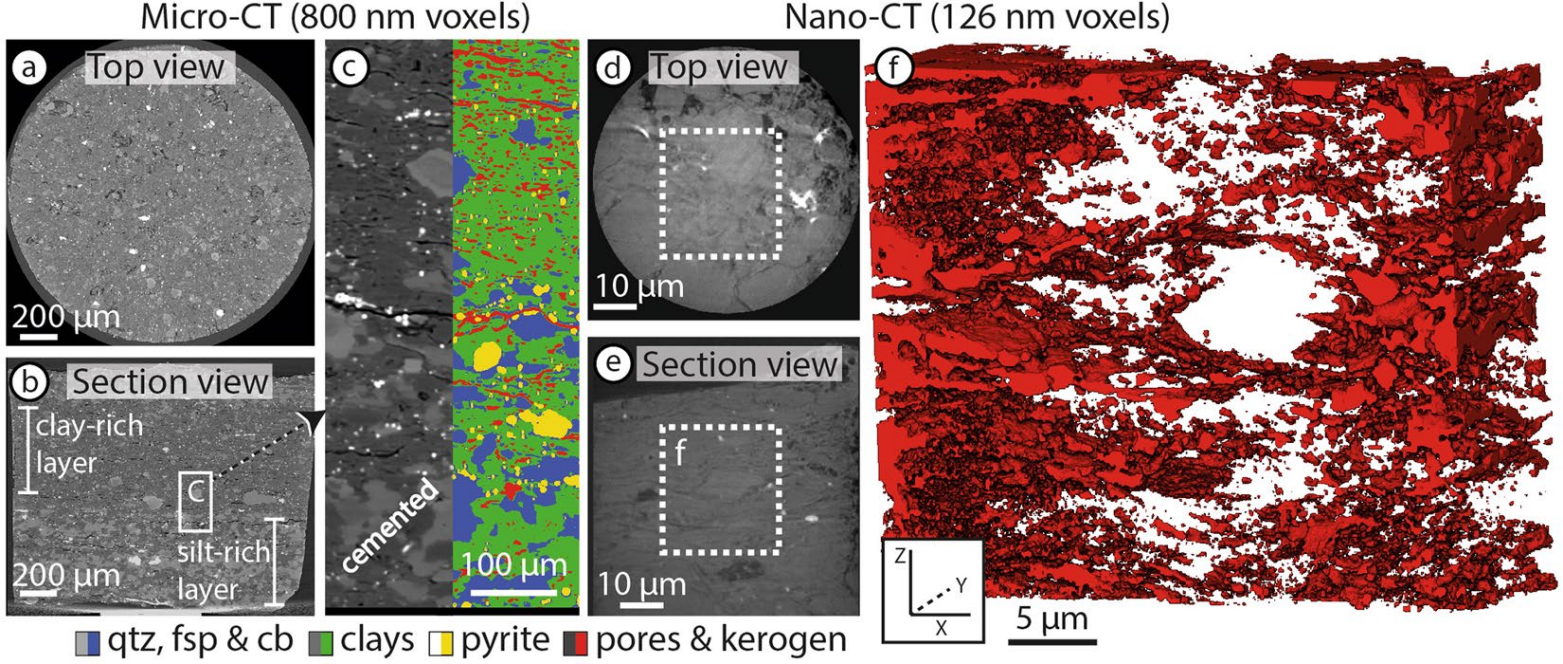
X-ray computed tomography data. (**a**–**c**) Zeiss Xradia 520 Versa micro X-ray computed tomography with voxel size of 0.8 microns. (**a**) Lamination-parallel view of sample. (**b**) Vertical section view through sample showing silty (7% porous phase) and clay-rich (13% porous phase) compositional laminations of approximately 0.5 mm thickness. (**c**) Segmentation of grayscale into 4 distinguishable phases. (**d**–**f**) Zeiss Xradia 810 Ultra nano X-ray computed tomography with voxel size of 0.126 microns. (**d**) Lamination-parallel view of sample. (**e**) Vertical section view through sample. (**f**) Porous volume rendering (dashed volume outline in (**e**)) of grayscale threshold segmentation representing 11 volume % (red).

A 2 mm tall by 2 mm diameter pillar of sample 4 was scanned using the Versa micro-CT at a voxel resolution of 800 nm (Fig. 2a–c). Four different phases are clearly segmented by their grayscale thresholds (Fig. 2c). The segmented phases include clastic grains (23%: quartz, carbonates and feldspars), clays (63%), pyrite (4%) and a low density porous phase (10%). The porous phase includes both physical pores and kerogen, which are difficult to distinguish from one another at this resolution. Compositional layering defined by clay-rich (top) and silt-rich (bottom) laminations is observed within the 2 mm tall pillar (Fig. 2b), with clay modal proportions of 70% and 52%, respectively. The modal proportions of the porous phase are 13% in the clay-rich layer and 7% in the silty layer. In the clay-rich layer the porous volume forms discontinuous, closely spaced wavy surfaces subparallel to the lamination. In contrast, the porous phase in the silty layer is less dispersed and concentrates as isolated lenses and pockets subparallel to lamination. The lower pore volume fraction is likely augmented by cementation around the silty grains, which do not show distinct grain boundaries (Fig. 2c) or expected inter-grain pores associated with coarser sediments^56,57^. A larger lamination-parallel fracture across the sample is associated with the silty layer (Fig. 2c). The micro-fracture spacing evident from the micro-CT is 50 to 400 *μ*m. We exported binary porous phase and bulk rock 3D sub-volumes of each layer for modelling of the pore phase interconnectivity.

A 64 *μ*m tall by 64 *μ*m diameter pillar of sample 2 was scanned twice using the Ultra nano-CT at a voxel resolution of 64 and 126 nm. The nano-CT scan illuminates the scaly fabric of the clay minerals wrapping around the clastic mineral grains (Fig. 2d–f). The compositional grayscale thresholds are more difficult to segment in the nano-CT results compared to the micro-CT, which stems from the lower energy used for nano-CT imaging and lower signal to noise ratio. However, we can use the porous phase volume percent from the micro-CT scan to segment a similar volume proportion in the nano-CT results (~10%, Fig. 2f). The high-resolution scan shows the sample preparation damage from the laser at the edge of the sample (right edge in Fig. 2e). We segmented the porous phase in a sub-volume from within the 3D data (~25 *μ*m^3^: dashed box in Fig. 2d,e) to avoid the laser damage. The sub-volume also excludes the base and circumference edge of the sample, which returned darker grayscales. This is due to both the source of Ultra showing a Gaussian beam profile distribution on the detector and the maximum intensity of X-rays is registered in the centre of the field of view, decreasing radially towards the edges/corners. Due to the less precise grayscale representation of porous phase volume in the nano-CT data, we segmented a range of pore phase volumes of 3, 5, 10 and 20% to compare changes in pore phase interconnectivity. This range represents the prospective range of pores and kerogen in shale gas plays^1^ and allows us to analyse the effect of changing the porous phase volume on permeability.

**Figure 3:**
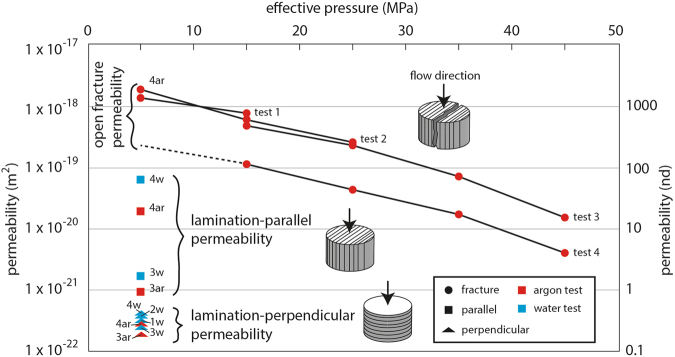
Experimental argon (red) and water (blue) permeability results through 20 mm diameter by 20 mm length shale cores. Permeability was measured perpendicular to laminations (triangles), parallel to laminations (squares) and along a lamination-parallel fracture (circles).

Permeability was measured at low effective pressures of 5 MPa to maximise the downstream pressure oscillation signal during experiments and to compare the relative directional permeability. The low effective pressure used in experiments allows us to better measure the very low matrix permeabilities, but these will scale to even lower permeabilities for deeper reservoir conditions^27^. However, it is easier to quantify the relative directional permeability at low effective pressures. The results are summarised in Table 1. For both water and argon experiments, the lamination-parallel permeability (k_*v*_) ranges from 1 × 10^−19^ to 1 × 10^−21^ m^2^ (Fig. 3, squares), whereas lamination-perpendicular permeability (k_*h*_) ranges between 1 × 10^−21^ and 1 × 10^−22^ m^2^ (Fig. 3, triangles). For each sample the permeability of argon is systematically lower than the permeability of water, but showing the same one to two orders of magnitude difference in directional permeability. The relative permeability of argon and water, may be affected by fluid mixtures present within the sample during the experiment, which can lower the effective permeability^26,27^.

One core plug from sample 4 that split along lamination was tested for argon permeability at varying confining pressure conditions and the results are summarised in Table 2. The permeability through this sample is 1 × 10^−18^ m^2^ at 5 MPa effective pressure and the permeability decreases by two orders of magnitude to 1 × 10^−20^ m^2^ with increasing effective pressure to 45 MPa (Fig. 3 circles, test 1–3). After reloading the sample with the lamination-parallel fracture for a 4th test, the permeability results were lower, but with the same 2 order of magnitude change with increasing effective pressure (Fig. 3, test 4). This difference may result from a tighter fit of the two halves during the re-assembled of the sample for test 4.

**Table 2:** Argon gas permeability was measured by the pore pressure oscillation technique through an open fracture parallel to lamination of sample 4.

Effective pressure (MPa)	5	15	25	35	45
test 1 (m^2^)	1.4 × 10^−18^	7.8 × 10^−19^			
test 2 (m^2^)	1.9 × 10^−18^	6.3 × 10^−19^	2.5 × 10^−19^		
test 3 (m^2^)		4.7 × 10^−19^	2.2 × 10^−19^	7.4 × 10^−20^	1.6 × 10^−20^
test 4 (m^2^)		1.1 × 10^−19^	4.4 × 10^−20^	1.8 × 10^−20^	4.0 × 10^−21^

Measurements were taken continuously while the confining pressure was increased at 10 MPa increments from 10 MPa to 50 MPa. Pore pressure was set at 5 MPa with a 1 MPa oscillation amplitude at 1 hour wavelengths. The sample was left in the apparatus overnight at 5 MPa effective pressure between tests 1, 2 and 3. The sample was extracted from the apparatus after test 3 and reloaded for a 4th test.

### Interconnectivity of porous phase

Anisotropic permeability behaviour of shales is well known and expected from the strong mineral alignment and the macro-scale bedding and lamination fabric. In this section we explore the available permeable pathways through the porous phase volume (kerogen and pores) at the micron scale. The three-dimensional distribution of the porous phase is relatively homogenous across the millimetre scale in two dimensions (Fig. 1), but shows a textural and modal variation across compositional layering of clay-rich and silt-rich laminations (Fig. 2c). In the tests below we also look at the compositional and mineralogical effect on interconnectivity.

#### 2D Image Analysis

**Figure 4:**
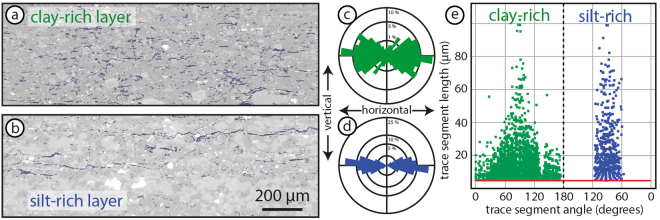
Two-dimensional fracture analyses using FracPaQ^58^. Clay-rich layer (**a**) and silt-rich layer (**b**) from sample 2 with X-ray CT image overlain with FracPaQ image analyses from binary porous phase image. Orientation rose diagram (**c**,**d**) and length versus angle (**e**) results for each layer (clay-rich layer = green; silt-rich layer = blue).

The FracPaQ^58^ fracture analysis tool for MATLAB is designed to characterise linear features, such as fractures. This tool can be used on any two-dimensional data to quantify linear features. We used the FracPaQ script to analyse the two-dimensional interconnectivity and alignment of the porous phase in clay-rich shale samples. Binary images of the porous phase for the clay-rich and silt-rich layers are converted from the micro-CT data and imported into the script separately for each layer (Fig. 4a,b). The alignment of the porous phase in the clay-rich layer is anastomosing subparallel to the laminations, forming a well defined scaly fabric (Fig. 4c). In contrast, the orientation of interconnected pathways in the silty layer are restricted to within ±30° around the lamination plane (Fig. 4d). The interconnected trace lengths of the porous phase exhibits a weakly defined directional preference of longer trace segments parallel to the lamination (90°, Fig. 4e). In both layers the interconnected porous phase segments in two dimensions are up to 100 *μ*m long with the majority shorter than 40 *μ*m (Fig. 4e). The results discard disconnected/isolated segments of less than 5 microns in length (red base line in Fig. 4e).

#### 3D Tortuosity Factor

**Figure 5:**
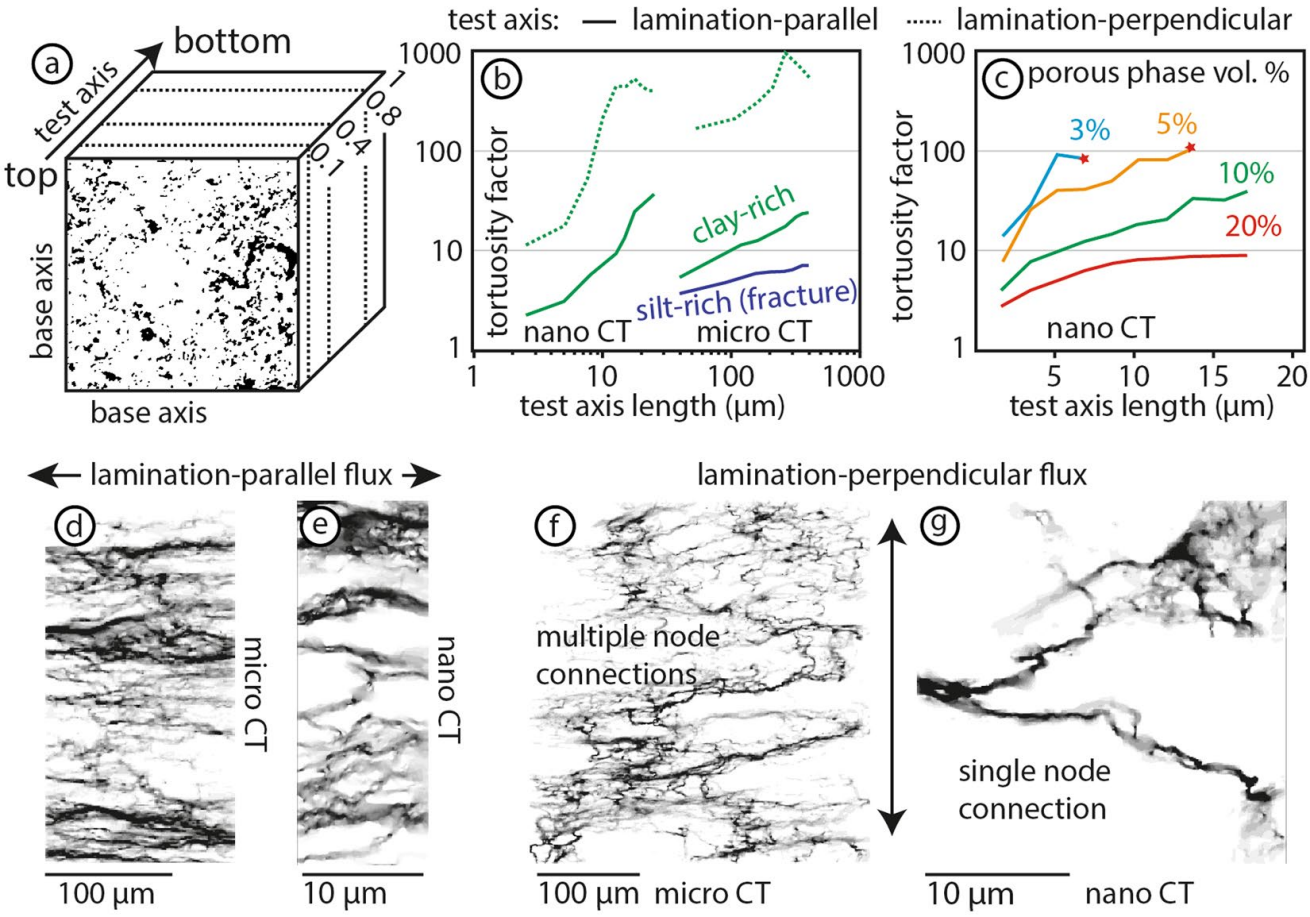
TauFactor^48^ three-dimensional tortuosity factor analyses. Detailed results are provided in the supplementary material (Table S1). (**a**) Section of binary volume (black = porous volume; white = non-porous volume) in a schematic of tortuosity factor analysis with constant base axes and increasing the unit length of the test axis at 10% proportions. (**b**) Plot of average tortuosity factors for nano- and micro-CT volumes. (**c**) Porous phase volume percent comparison from 3 to 20% using the nano-CT volume along the bedding-parallel axes. Red star at the end of 3 and 5% tests is the point at which the computation failed. (**d**–**g**) Stacked 3D image of modelled interconnected pathways through clay-rich layer at micro- and nano-scale.

We use TauFactor^48^ to model the three-dimensional interconnectivity of porous phase volumes segmented from micro- and nano-CT. The full results are provided in the supplementary material (Table S1). TauFactor is a MatLab application for simply calculating the reduction in diffusive transport caused by convolution in the geometry of heterogeneous media. Tortuosity factor quantifies the geometric interconnectivity of pore space, taking into account the changing cross-sectional area of pores^46,47^. TauFactor calculates the directional tortuosity factor (*τ*) along three mutually perpendicular axes of interconnected “diffusive phases” (or porous phase) through a three-dimensional volume. The minimum value of tortuosity factor for is 1, which would represent a perfectly straight pathway of equal cross-sectional area running parallel to the test axis. TauFactor also calculates the representative volume element (RVE) and computes each directional tortuosity factor at set proportions of the volume, starting with 10% length of the volume, then 20% and so on (Fig. 5a). In a homogenous system the tortuosity factor value tends towards a maximum value with an increasing length unit of the test axis^39^, if the mineral fabric, size and distribution of the sample are representative of the system. We computed the tortuosity factor using the equal area method in TauFactor (A = “constant from top/bottom”), keeping the base area of the two axis perpendicular to the tested axis direction constant (Fig. 5a). If there are no interconnected pathways across the tested volume, the computation fails and TauFactor returns a value of infinity. Due to the heterogeneity of mineral distributions in natural shales, the tortuosity factor will vary from sample to sample. However, the small mineral grain sizes of our shale samples (<25 micron, Fig. 1) allow for meaningful tests of mineralogical control on flow paths to be examined by high-resolution X-ray CT (Fig. 2).

In each model, the tortuosity factor increases as the test axis length increases (Fig. 5b). The micro-CT volume was modelled for each compositional layer representing 13% porous phase in the clay-rich layer and 7% porous phase in the silt-rich layer (Fig. 2b). The upper clay-rich volume has a tortuosity factor of ~25 in both lamination-parallel orientations over an axis-length of 400 microns. Interconnected pathways along laminations in the silty layer are predominantly in and around the fractures and organic matter lenses resulting in a lower tortuosity factor below 10, representing shorter pathways across the test-axis. Nano-CT results are computed for a 3, 5, 10 and 20% porous phase volume. Lamination parallel tortuosity factor for all measurements fall within the same order of magnitude as the micro-CT results, although there are small variations between the two mutually perpendicular directions parallel to the lamination (see supplementary Table S1 for details). The low tortuosity factor values for short test axis lengths below 5 *μ*m correlate to short, straight segments below the mineral-scale (Fig. 2e). For both the nano- and micro-CT volumes, the tortuosity factor computed perpendicular to laminations is an order of magnitude higher in the clay-rich layer (dotted lines, Fig. 5b). No vertical interconnectivity is computed for the silty layer with the lower porous phase volume (tortuosity factor of infinity).

We compared tortuosity factor results for different volume percentages of the porous phase from 3 to 20% for the same nano-CT volume by manually changing the grayscale threshold (Fig. 5c). The interconnectivity of 3% porous phase volume failed at less than 9 *μ*m unit length and below ~14 *μ*m for 5% in both lamination-parallel directions (stars in Fig. 5c). At 10% and above there is generally continuous interconnectivity across the whole sample in the lamination-parallel direction (Fig. 5c). No lamination-perpendicular interconnectivity longer than 4 *μ*m unit length is computed at the mineral scale for the low porous phase volume of 3 and 5%. At 10% porous phase volume, the interconnected pathways reached up to 13 *μ*m unit length across laminations for Ultra CT scans with a resolution of 126 nm. The higher resolution scan of 63 nm computed an interconnectivity at 10% porous phase volume through the sample to over 20 *μ*m (see supplementary Table S1 for details). In our simulations, the most consistent lamination-perpendicular interconnectivity across the whole sample (20 *μ*m) occurs for porous phase volumes of more than 20%. Increasing the porous phase volume has two effects on the interconnected pathways through the sample: (1) The tortuosity factor decreases and (2) the interconnected length increases (Fig. 5c).

In both lamination-parallel and lamination-perpendicular models, the tortuosity factor is higher through 20 microns of the nano-CT sample than through 40 microns of the micro-CT sample (Fig. 5b). Scaling of statistically self-similar objects, in this case the interconnectivity of the porous volume, is well know. For example, the length of Britain’s coast is inversely proportional to the length of the yardstick used to measure it^59^. Similarly, tortuosity factor also scales inversely with voxel size. However, the higher resolution of the nano-CT is expected to reveal more pores compared to the micro-CT and consequently the tortuosity factor should be reduced. The tortuosity factor at the nano-scale will be highly dependant on the mineral geometry of the scanned volume, such as the distribution and modal proportion of clay- and silt-sized grains. We can explain decrease in tortuosity factor with the step up in scale into the micro-CT experiment by the amount of available nodes that connected different segments in the volume across the set base area of the volume. The volume we imaged with nano-CT only had one node that interconnected the porous phase in the lamination-perpendicular direction (Fig. 5g). Increasing the base area perpendicular to the directional test-axis increases the available nodes that interconnect adjacent pathway segments (Fig. 5f). This scaling leads to a decrease of the tortuosity factor, as more direct pathways parallel to the test axis become available as more nodes are introduced to the tested volumes.

## Discussion

**Figure 6:**
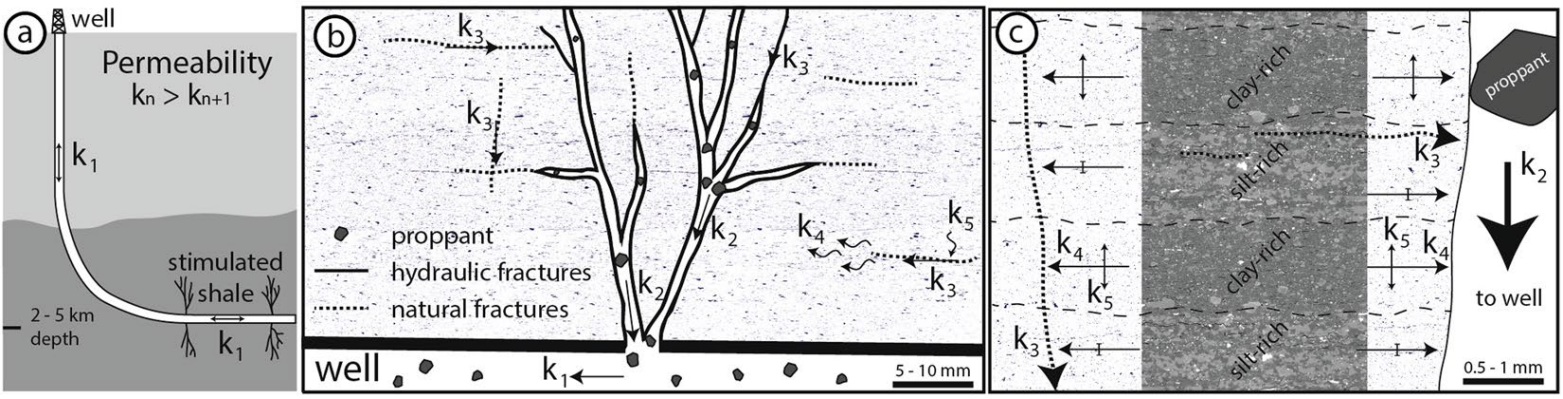
Permeability hierarchy of shale gas wells from well to clay minerals. (**a**) Well access (k_1_) to shale gas reservoir with stimulated hydro-fracture network. (**b**) Propped (k_2_) and unpropped (k_3_) fracture network connected to well with mm-scale pore distribution background from QEMSCAN results. (**c**) Inter-fracture lamination and mineral-scale fabric-controlled permeability (k_4&5_) with micron-scale pore distribution background from QEMSCAN results and mineral distribution scans from micro-CT results.

Ultimately, the rate of shale gas recovery during the production life of wells is determined by the permeable structure that exist at depth around the stimulated borehole (Fig. 6a). Over-pressured fluids with proppants are injected into the reservoir to open up natural fractures and induced hydro-fractures to increase the formation permeability and allow for trapped gas to escape through propped open pathways (Fig. 6b). The longevity of shale gas recovery reported from operating wells, i.e. the decline curve^9,10^, records the interconnectivity of hierarchical permeabilities through fractures with and without proppants, and the inter-fracture matrix within the stimulated area (Fig. 6c).

The results presented in this study combine three-dimensional and two-dimensional geometric and interconnectivity analyses of porous phases with physical permeability measurements in the laboratory. The measurements and models characterise the relative fracture, lamination-parallel and lamination-perpendicular flow through clay-rich shales. At low effective pressures of 5 MPa, we record directional permeabilities of 1000 nano-darcy (nd) through fractures (k_3_), 10 to 100 nd parallel to laminations (k_4_) and 0.1–1 nd across clay-rich laminations (k_5_, Fig. 6b,c). The anisotropic behaviour of directional matrix-permeability through the samples is recorded using both water and argon (Fig. 3), consistent with a fundamental geometric control under controlled and constant effective pressures. Our results show the same one to two order of magnitude anisotropy in directional permeability relative to the sedimentary laminations that is commonly reported for shales^31,60,61^. Permeability along a fracture increases by another one to two orders of magnitude, relative to lamination-parallel matrix permeability. The fracture permeability scales down by two orders of magnitude with increasing effective pressures to 45 MPa, simulating typical reservoir depth conditions (Fig. 3). The same relative order of magnitude differences are computed at the micron scale by the directional tortuosity factor through three dimensional reconstructions of the same samples used in physical experiments (Fig. 5b).

The effective permeability at which fluids are transported through tight shales is a combination of hydraulic and diffusive flow through porous media, made up of micro- and nano-pores within the kerogen and clay matrix^18,21,22^. Hydraulic permeability (k) is generally defined in terms of tortuosity (*τ*, equation 1):

1 $$k=\frac{{\phi }^{3}}{c{\tau }^{2}{S}^{2}},$$

where k is permeability given by the porosity (*ϕ*), the Kozeny constant (c) that depends on the pore shape, and the specific surface area (S) around the pores^39,62^. Effective diffusivity (*D*_*eff*_) is given below in terms of tortuosity factor (τ^*^, equation 2):

2 $${D}_{eff}={D}_{0}\frac{\varepsilon }{{\tau }^{* }},$$

where *D*_0_ is the phase diffusivity and *ε* is the volume fraction of the porous media^48,63^. In equation 1, the permeability is inversely proportional to tortuosity and the shape of the pores. Variations in pore shape, or cross-sectional area of flow paths, are incorporated into the three-dimensional calculations of tortuosity factor. Therefore, hydraulic and diffusive flow are both inversely proportional to the tortuosity factor (and tortuosity).

Collecting high resolution images that clearly differentiate kerogen and nano-pores is possible, but difficult to achieve^24^. Our CT scans were unable to segment kerogen and pores and, therefore, we modelled the interconnectivity of both as a single porous phase (*ε*, see Fig. 2d–g). This simplification means that our method misses the ultra-fine nano-pores observed in kerogen^37^ and that *ε* (pores and kerogen, equation 2) is greater than *ϕ* (porosity only, equation 1). However, the nano-porous flow is thought to have restricted diffusive flow because of the tighter constrictions of the pore walls^29^ and will occurs within the kerogen proportion of the volume imaged by the X-ray CT. Although nano-porous transport will add significant insight into modelling relative flow mechanisms, estimating diffusive flow across whole kerogen volumes allows us to integrate the nano- and micro-scale pore network and compare the directional interconnectivity of flow pathways at a representative scale for shales. Our results allow for the first direct comparisons of three-dimensional pore space distributions with laboratory experiments of the same samples, where we measure permeability across a sample that combines both hydraulic permeability and diffusive flow^53^, the proportion of each dependent on the distribution of the different materials that control the flow mechanisms.

Both X-ray CT and QEMSCAN imaging show a predominantly homogenous and diffuse distribution of the porous phase throughout the sample. This implies that *ε* (and *ϕ*) are relatively constant across each sample. We can assume that *D*_0_ is also constant for each sample, because the relative proportion of kerogen and pore space probably remain similar for the small sample sizes used in both experimental and imaging techniques. However, these parameters will vary with different mineral compositions of shales and rocks in general, which affect the geometry and proportion of porous phase distributions. All the samples used in this study have the same modal proportion of clay (~60%), which defines the bulk of the rock volume and the rock texture. The interconnectivity of porous media in both hydraulic and diffusive flow is a function of the pore geometry^64,65^, which is inherited from the mineralogy. Therefore, our results show that the anisotropic permeability behaviour of ~60% clay-rich shales is fundamentally controlled by the tortuosity (or tortuosity factor) of interconnected porous phase volumes through a scaly clay fabric.

It is well known that tortuosity tends towards 1 as the porous phase volume increases towards 100 percent, although the tortuosity is only reported for porous phase volumes above 10%^62,66^. In two-dimensional image analyses the interconnection of pores in our shale samples is up to 100 *μ*m in length and rarely extends above 40 *μ*m, even along fractures (Fig. 4e). Therefore, it is necessary to reconstruct the three-dimensional porous phase distribution to accurately simulate flow. We tested the effect of varying the porous phase volume manually within the same bulk volume from 3 to 20% (Fig. 5c). Higher volumes resulted in longer interconnected pathways with a lower tortuosity factor, consistent with our understanding of the porosity-tortuosity relationship. Our results show that the critical porous phase volume required for any significant lamination-parallel flow interconnectivity through the clay-rich shale is between 5 and 10%. Continuous interconnectivity across laminations requires higher porous phase volumes (≥20%) than in the lamination-parallel direction to achieve interconnectivity. There is no systematic variation in the two mutually perpendicular directions within the plane parallel to laminations, but this will be affected by local variations in kerogen and pore space distribution in a natural system.

We compared the compositional effect of silt-rich (52% clays) and clay-rich (70% clays) layers using the micro-CT results with a porous phase volume of 7% and 13%, respectively. Due to the lower clay fraction in the silty layer there are predominantly planar interconnections in porous phase volumes along fractures and kerogen lenses (Fig. 4d). No vertical interconnectivity is computed across the silty layer. The scaly geometry of the porous phase following the clay geometry in the clay-rich layer (Fig. 4c) leads to a higher potential of cross-lamination interconnected pathways through the clay network (Fig. 5f,g). In general, more the silt-rich rock compositions are more rigid have a higher fracturing potential, which is sought after during hydro-fracturing^67,68^, but at the scale of compositional laminations the silty layers form barriers to layer-perpendicular flow, especially with lower porous phase volumes due to cementation (Fig. 6c). Ultra-low permeability measured in experiments on these same samples suggests that some interconnected pathways are available at the cm-scale (triangles, Fig. 3). This is likely enhanced by decompression fractures that concentrate around silty layers (Fig. 1). The quantity of the porous phase volume has a strong affect on interconnectivity (Fig. 5c). Therefore, small fractures or isolated permeable pockets, which increase the sub-mm porous phase volumes will enhance the ultra-low permeability at the cm-scale. As shown, the mineralogy of our sample set is dominated by clays. Our results show that the anisotropy of the directional permeability in clay-rich shales calculated from high-resolution imaging (*μ*m-scale) is of the same order of magnitude to measurements in physical experiments (mm-scale). Therefore, the macro-scale permeability structure through shales is effectively determined by the geometry of rock forming minerals, in this case the alignment of clays in between and around silt-sized grains.

The lowest tortuosity factor was calculated for lamination-parallel interconnectivity in the silty layer (<10, Fig. 5b). This low tortuous geometry stems from the fracture that cuts across the entire silty layer (Fig. 2c) and is comparable to the experimental permeability measurements through a lamination-parallel fracture (sample 4, Fig. 3). The lamination-parallel fractures observed in the samples and imaged by QEMSCAN (Fig. 1) and micro-CT (Fig. 2) are uncemented fractures that probably formed by decompression during drilling and retrieval of the samples from depth. Decompression fractures are closed at depth and lead to overestimations of the natural fracture density^68,69^. Nevertheless, the permeability results for the fractured sample provide an estimate for permeability through unpropped fractures (k_3_, Fig. 6), relative to the matrix permeability. In addition to the lower tortuosity factor of fractures, the effective permeability through fractures will be higher compared to the matrix, because the interconnected porous phase volume has a lower specific surface area (S, equation 1) for larger pore dimensions (*ϕ*, equation 1), leading to a smaller overall component of slower diffusive flow (*D*_*eff*_, equation 2). Research has shown this relationship, where the flow mechanism through open fractures is dominated by higher permeability hydraulic flow^19,70^. During hydro-fracturing of shale gas plays, the immediately accessible gas will be extracted by higher flow rates through stimulated fractures, followed by ultra-low permeability through the rock matrix that can access the fractures via a complex network of interconnected porous phases.

## Conclusions

Combining high-resolution X-ray CT imaging with physical permeability experiments illustrate the micron-scale interplay between directional matrix and fracture permeability through shales (Fig. 6). The homogenous and diffuse distribution of pore space and kerogen at the inter-fracture scale suggests that the longevity of shale gas recovery recorded in production decline curves is governed by the accessibility of gas trapped in the matrix. The detailed mineral-scale control on the anisotropic permeability behaviour of shales shown in this study indicates that the broader accessible resource is more likely to be laterally around the stimulated fractures network than vertically. Existing natural fracture networks play an integral role in defining the larger scale interconnectivity at depth and the immediately accessible shale gas recovered during peak production of wells. Recent experiments are showing that the productivity of shale gas wells is enhanced by using finer grained proppants (1 to 50 *μ*m), compared to typical proppants of 100 to 300 *μ*m in size used to stimulate unconventional gas reservoirs^71,72^. These “micro-proppants” infiltrate smaller natural and induced fractures within the stimulated zone and increase the shale-fracture interface area, which allows for more directly accessible gas to escape via the open fractures. These technical advances in shale gas recovery are explained by our detailed characterisation of the micro-scale porous phase distribution and permeability structure within the matrix of shales in between the fracture network, where the recoverable resource in the shale matrix is stimulated laterally around the fracture network.

## Electronic supplementary material

Supplementary Information

Supplementary Table S1
