# Author Correction: Quantifying the anisotropy and tortuosity of permeable pathways in clay-rich mudstones using models based on X-ray tomography

**DOI:** 10.1038/s41598-018-22646-6

**Published:** 2018-03-06

**Authors:** Nils R. Backeberg, Francesco Iacoviello, Martin Rittner, Thomas M. Mitchell, Adrian P. Jones, Richard Day, John Wheeler, Paul R. Shearing, Pieter Vermeesch, Alberto Striolo

**Affiliations:** 1 University College London, Department of Earth Sciences, London, WC1E 6BT UK; 2 University College London, Electrochemical Innovation Lab, Department of Chemical Engineering, London, WC1E 6BT UK; 3 Halliburton, Chiswick Park, London, W4 5YE UK; 4 University of Liverpool, Department of Earth, Ocean and Ecological Sciences, Liverpool, L69 3BX UK

Correction to: *Scientific Reports* 10.1038/s41598-017-14810-1, published online 01 November 2017

The original version of this Article contained a typographical error in the spelling of the author Thomas M. Mitchell, which was incorrectly given as Tom M. Mitchell. This has now been corrected in the PDF and HTML versions of the Article and in the accompanying Supplementary Information file.

