# Supplementary material for: Quantifying the anisotropy and tortuosity of permeable pathways in clay-rich mudstones using models based on X-ray tomography
Source: Sci Rep. 2017 Nov 1;7:14838. doi: 10.1038/s41598-017-14810-1 (PMC5665904; doi:10.1038/s41598-017-14810-1)
Supplement: Supplementary file 1 — Supplementary Information [file 41598_2017_14810_MOESM1_ESM.docx]

# Quantifying the anisotropy and tortuosity of permeable pathways in clay-rich mudstones using models based on X-ray tomography: Supplementary Information Table S1

Nils R. Backeberg^1^, Francesco Iacoviello^2^, Martin Rittner^1^, Thomas M. Mitchell^1^, Adrian P. Jones^1^, Richard Day^3^, John Wheeler^4^, Paul R. Shearing^2^, Pieter Vermeesch^1^, Alberto Striolo^2^

[1] University College London, Department of Earth Sciences, London, WC1E 6BT, UK

[2] University College London, Department of Chemical Engineering, London, WC1E 6BT, UK

[3] Halliburton, Chiswick Park, London, W4 5YE, UK

[4] University of Liverpool, Department of Earth, Ocean and Ecological Sciences, Liverpool, L69 3BX, UK

We submit a table of tortuosity factor results produced from TauFactor as supplementary information with the submitted manuscript. The table data reports tortuosity factor at each representative volume element (RVE) directional test axes. We provide the data for each directional test at a given resolution and representative porous phase volume (Supplementary Table S1). The Ultra CT data was compared for two opposite directions along the same axis (from top, from bottom), to test variability of computations for the same volume fraction. Although the intermediate tortuosity factor calculations (RVE < 1) varied along the axis with opposite starting points, the final volume tortuosity was the same (at RVE = 1).
